# Liddle Syndrome Presenting as Hypertensive Crisis and Myocardial Injury in a Young Male: A Case Report

**DOI:** 10.1155/crin/7218869

**Published:** 2026-06-28

**Authors:** Abbas Khan, Alina Batool, Askar Nisar, Jaweria Farman, Okasha Tahir, Muhammad Hassaan Javaid, Raghabendra Kumar Mahato, Tayyeb Ali

**Affiliations:** ^1^ Department of Cardiology, Hayatabad Medical Complex, Peshawar, Khyber Pakhtunkhwa Province, Pakistan, hmcpeshawar.com.pk; ^2^ Department of Nephrology, Khyber Girls Medical College, Peshawar, Khyber Pakhtunkhwa Province, Pakistan; ^3^ Department of Medicine, Ayub Medical College, Abbottabad, Khyber Pakhtunkhwa Province, Pakistan, ayubmed.edu.pk; ^4^ Department of Medicine, Khyber Medical University, Peshawar, Khyber Pakhtunkhwa Province, Pakistan, kmu.edu.pk; ^5^ Department of Medicine, Shifa College of Medicine, Islamabad, Pakistan; ^6^ Department of Nephrology, Gomal Medical College, Dera Ismail Khan, Khyber Pakhtunkhwa Province, Pakistan, gmcdikhan.edu.pk; ^7^ Department of Medicine, Gandaki Medical College Teaching Hospital and Research Center, Pokhara, Gandaki Province, Nepal

## Abstract

**Introduction:**

Liddle’s syndrome is a rare inherited disorder associated with early‐onset and resistant hypertension due to hyperactivity of epithelial sodium channels (ENaCs). The condition is caused by mutations in the genes SCNN1A, SCNN1B, and SCNN1G. Liddle’s syndrome leads to hypertension, hypokalemia, and metabolic alkalosis.

**Case Presentation:**

A previously healthy 21‐year‐old male patient complained of severe central chest pain radiating to both arms and had a hypertensive emergency with a BP reading of 267/170 mmHg. The patient had marked elevation in troponin I (13.9 ng/mL), hypokalemia (2.8 mmol/L), and metabolic alkalosis. The electrocardiogram showed left ventricular hypertrophy with a strain pattern and QTc prolongation due to hypokalemia. Coronary angiography ruled out the possibility of primary acute coronary syndrome by revealing normal coronary arteries. Echocardiogram showed concentric left ventricular hypertrophy with normal ejection fraction and no wall motion abnormalities. In search of the secondary cause of hypertension, renal Doppler ultrasonography and aldosterone‐to‐renin ratio were normal, thus ruling out primary hyperaldosteronism. About the typical clinical and biochemical presentation, a clinical diagnosis of Liddle syndrome was highly likely. Genetic tests were not performed owing to financial constraints. However, treatment with amiloride brought about remarkable improvement in the control of hypertension and normalization of potassium levels.

**Conclusion:**

Liddle’s syndrome should be suspected in cases where there are young individuals who have severe hypertension, hypokalaemia, and an aldosterone‐to‐renin ratio that is normal or low. The diagnosis must be made promptly because delay may lead to complications. In instances where molecular testing is impractical, treatment with ENaC inhibition using amiloride is diagnostic and potentially life‐saving.

## 1. Introduction

Liddle syndrome is an unusual type of secondary hypertension caused by autosomal dominant gain‐of‐function mutations in genes for ENaC subunits. It commonly presents in childhood or young adulthood; however, some patients’ disease is not diagnosed until they are middle‐aged [1, 2].

On the molecular level, the majority of disease‐causing mutations occur in the PPPXY consensus sequence, known as the PY motif, which is part of the cytosolic tail region of the beta or gamma subunit of ENaC [1–3]. This sequence is crucial for interaction with the E3 ubiquitin ligase enzyme Nedd4‐2, which normally ubiquitinates ENaC subunits, causing internalization and proteolytic degradation of the channel from the plasma membrane. As a result, there is a limited number of ENaCs in the apical membrane. Loss‐of‐function or deletion mutations impair Nedd4‐2’s activity and lead to overexpression of constitutively active ENaC channels on the apical membrane. This allows for excessive sodium reabsorption, causing extracellular volume expansion, decreased secretion of renin, and increased renal potassium and hydrogen ion wasting due to negative luminal transmembrane potential difference, leading to a combination of hypertension, hypokalemia, and metabolic alkalosis [4, 5].

The above pathophysiologic cascade leads to a phenotype of mineralocorticoid excess in the complete absence of an aldosterone increase. This explains why the disease has been alternatively named pseudohyperaldosteronism. Differentiating features from primary hyperaldosteronism, which manifests with renin suppression and an increase in aldosterone levels, is vital in diagnosis. Only about 100 families or sporadic cases have been described globally since its initial reporting by Liddle [3].

In the subgroup of causative genes, mutations in SCNN1B (β‐ENaC subunit) and SCNN1G (γ‐ENaC subunit) account for the majority of cases, while those in SCNN1A are very uncommon. Clinical description of genetically proven patients indicates that ENaC‐mediated hypertension and family history of hypertension are the most common symptoms, although penetrance, severity, and onset age differ widely, even among individuals from the same family line. Notably, the degree of clinical variation is apparently not dependent on any particular type of SCNN1 gene or variant [6].

The treatment of choice involves the use of ENaC inhibitors, such as potassium‐sparing diuretics amiloride or triamterene, alongside a low‐sodium diet to normalize blood pressure and fix electrolyte disturbances. Traditional RAAS‐based treatment strategies do not work well because the basic problem lies upstream of aldosterone signaling. The disease can also be diagnosed on the basis of treatment response and genetic confirmation, if possible [7, 8].

This paper discusses a case of a 21‐year‐old male patient with hypertensive crisis, hypokalemic metabolic alkalosis, and raised cardiac troponins who was eventually diagnosed with Liddle syndrome after ruling out all other secondary causes of hypertension. This case highlights the need for thorough investigation of hypertensive patients, especially the younger ones. It also underscores the utility of an ENaC blockade as a diagnostic tool in areas with limited resources where genetic studies cannot be performed.

## 2. Case Presentation

A 21‐year‐old premorbidly healthy male presented with acute‐onset severe central chest pain radiating to both arms and associated with diaphoresis. On presentation, his blood pressure was 267/170 mmHg, consistent with a hypertensive emergency. His heart rate was 100 beats/minute, while oxygen saturation and respiratory rate were within normal limits. Cardiovascular examination revealed a loud second heart sound and a sustained, laterally displaced apex beat without audible murmurs or gallops.

The ECG revealed sinus tachycardia with a Sokolow–Lyon index voltage that satisfied the condition of LVH (S1 + RV5 = 38 mm). The lateral leads I, aVL, V5–V6 displayed depression in the ST segment and inversion of T waves, which were characteristic of an LVH strain pattern. The QT interval was prolonged due to severe hypokalaemia (QTc 470 ms). There were no ST elevations, signs of right bundle‐branch block, or ischemic Q waves.

The initial biochemical results showed an extremely high level of high‐sensitivity cardiac troponin I (13.9 ng/mL), potassium (2.8 mmol/L), and arterial blood gases, indicating metabolic alkalosis (pH 7.52, bicarbonate 30 mEq/L, and partial pressure of carbon dioxide 42 mmHg). Given the presence of raised troponins, severe chest pain, and electrocardiogram abnormalities, the patient underwent emergent coronary angiography that showed completely normal coronary arteries with no evidence of any obstruction, thereby ruling out ACS as the cause of myocardial damage.

TTE was performed subsequently and revealed concentric left ventricular hypertrophy (interventricular septum thickness 13 mm, posterior wall thickness 13 mm, and LV mass index corresponding with severe left ventricular hypertrophy), with normal left ventricular ejection fraction at 63%. There were no abnormal regional wall motion defects found. The examination of the valvular apparatus showed no abnormalities. Pericardial effusion was absent. Grade 1 diastolic dysfunction (pattern of impaired relaxation) was diagnosed based on tissue Doppler imaging results. Overall, these findings suggested hypertensive heart disease due to prolonged pressure overload, not cardiomyopathy or an infiltrative process.

Identification of hypertensive‐mediated myocardial injury with concentric LVH in the absence of obstructive coronary artery disease pointed to the investigation of other potential causes of hypertension. Clinical features of severe hypertension, hypokalemia, and metabolic alkalosis in an otherwise healthy young individual demanded a thorough evaluation of the cause of the disorder.

Doppler ultrasound of the renal arteries indicated a normal kidney size (right kidney 10.8 cm, left kidney 11.0 cm), normal biphasic waveform pattern in both arteries, and no renal artery stenosis or renal artery asymmetry, hence ruling out renovascular hypertension. The endocrine study showed a plasma aldosterone level of 36.80 ng/dL and plasma renin activity of 5.31 ng/mL/h, giving an ARR value of 6.93, which is within normal limits. The patient’s test results ruled out primary hyperaldosteronism, since this disorder exhibits increased aldosterone levels with decreased renin activity.

A thorough assessment for other possible secondary causes was performed. There were no clinical signs of Cushing’s syndrome, such as the absence of central obesity, dorsocervical fat pad, purple striae, proximal myopathy, or moon face. Pheochromocytoma was not considered because there was no history of episodic hypertension, sweating, headaches, or palpitations. There was also no history of ingestion of liquorice, carbenoxolone, exogenous mineralocorticoids, or other substances that may lead to perceived mineralocorticoid excess. There was no history consistent with Gordon syndrome (pseudohypoaldosteronism Type II), characterized by hyperkalemia and not hypokalemia.

A constellation of biochemistry, including hypertension, hypokalemia, metabolic alkalosis, and a normal ARR, was suggestive of Liddle syndrome, an uncommon autosomal dominant channelopathy associated with ENaC overactivity. A clinical diagnosis of Liddle syndrome was considered highly likely. Genetic testing for SCNN1B/SCNN1G genes was suggested as the definitive test; however, budgetary limitations prevented genetic testing during hospitalization. An ENaC inhibitor trial under strict clinical surveillance is a validated alternative diagnostic strategy in such resource‐limited environments when genetic testing is not feasible.

The patient was started on amiloride at 5 mg/day. Although his antihypertensive treatment was started with losartan and nebivolol, his blood pressure was consistently above 200 mmHg until the addition of amiloride to his treatment. After that, his blood pressure started to reduce gradually, while the normalization of his serum potassium concentration took place within 1 week. The patient was discharged when he was stabilized with amiloride at 10 mg/day, losartan (Tab Avsar), and nebivolol (Tab Nebix) along with his antiplatelets (Tab Lowplat Plus) and statins (Tab Rovista). His blood pressure measurements are shown in Table 1 below.

**TABLE 1:** Blood pressure monitoring chart.

Date	SBP (mmHg)	DBP (mmHg)	HR (bpm)	Medication and comments
Oct 28, 2025	267	170	100	Hypertensive emergency. Admitted. Tab Nebix + Tab Avsar initiated.
Oct 29, 2025	240	155	95	Inpatient monitoring. K^+^ 2.8 mmol/L.
Oct 30 2025	220	145	92	Diagnostic workup ongoing (Doppler, endocrine panel).
Oct 31, 2025	210	140	90	Liddle syndrome confirmed clinically. Amiloride 5 mg/day added.
Nov 01, 2025	185	125	88	Discharged. Amiloride 5 mg + Avsar + Nebix.
Nov 03, 2025	170	115	85	First outpatient review. Patient reports fatigue.
Nov 05, 2025	160	105	82	Amiloride dose uptitrated to 10 mg/day.
Nov 07, 2025	155	100	80	Serum K^+^ normalized.
Nov 09, 2025	148	95	78	Symptoms improving; no new complaints.
Nov 11, 2025	138	88	76	BP approaching target. Good compliance confirmed.
Nov 13, 2025	128	82	75	BP target achieved. Stable on amiloride 10 mg + Avsar + Nebix.

*Note:* Patient: 21‐year‐old male | target BP: < 130/80 mmHg.

## 3. Discussion

This particular case emphasizes the significance of exploring the secondary causes of hypertension among young patients with hypertensive emergencies and biochemical anomalies. While concentric hypertrophy of the left ventricle is expected among patients with chronic severe hypertension, the association of severe hypertension, hypokalemia, metabolic alkalosis, and a normal aldosterone‐to‐renin ratio warranted further workup in the form of genetic testing for a possible monogenic cause. The current case study shows that Liddle syndrome can mimic acute coronary syndrome in terms of chest pain, ECG changes, and elevated cardiac biomarkers, despite no evidence of obstructive coronary artery disease [9].

The diagnosis of Liddle syndrome in this case study depended mainly on biochemical correlation. In young patients who have severe or resistant hypertension, the presence of hypokalemia and metabolic alkalosis should lead to a search for secondary causes of hypertension. Plasma measurements of aldosterone and renin are essential in distinguishing diseases involving mineralocorticoid excess. In the current patient, the presence of a normal aldosterone‐to‐renin ratio ruled out primary hyperaldosteronism and shifted attention to diseases with excessive activity of ENaC independent of aldosterone action.

Pseudohyperaldosteronism or Liddle’s syndrome is a rare genetic disorder that follows an autosomal dominant inheritance pattern and is caused by gain‐of‐function mutations in genes coding for ENaC subunits (SCNN1A, SCNN1B, or SCNN1G) [10–12]. On the molecular level, these mutations act through the disruption of the PY motif (PPPXY) present in the cytoplasmic tail of the β‐ or γ‐ENaC subunit, which functions as the docking site for the E3 ubiquitin ligase, Nedd4‐2. Normally, Nedd4‐2 ubiquitinates ENaC subunits, thereby promoting their internalization and subsequent degradation by the proteasome or lysosome. Inactivation of the PY motif prevents the ubiquitination of the ENaC subunits by Nedd4‐2, leading to excessive expression of the ENaC subunits at the apical membrane. This causes unregulated sodium uptake, which leads to volume retention, inhibition of the renin–angiotensin–aldosterone system, and renal potassium loss [4, 5].

The following exclusionary analysis was performed to rule out other underlying causes for the noted biochemical triad of hypertension, hypokalemia, and metabolic alkalosis in the young patient. A series of disorders may be considered in the differential diagnosis of hypokalemic hypertension with renin suppression or renin deficiency [13]. Hyperaldosteronism (Conn’s syndrome) could be ruled out because of the normal aldosterone to renin ratio (ARR 6.93), whereas the latter condition would feature high levels of aldosterone and low renin concentrations [14]. Renovascular hypertension, resulting from renal artery stenosis, could be excluded based on the normal Doppler findings with preserved biphasic flow patterns in both kidneys [15]. Cushing’s syndrome caused by glucocorticoid excess was ruled out clinically, as no signs of hypercortisolism were present, such as central obesity, skin striae, or proximal muscle weakness [16]. Pheochromocytoma, a rare cause of hypertension, was considered less likely due to the lack of typical symptoms, including paroxysms, headaches, and sweating. Apparent mineralocorticoid excess (AME) resulting from 11β‐hydroxysteroid dehydrogenase Type 2 deficiency (either primary or secondary due to liquorice intake) was ruled out because of the lack of associated history [17]. Gordon syndrome (pseudo‐hyperaldosteronism Type II, also known as familial hyperkalemic hypertension) was excluded due to hypokalemia, not hyperkalemia. The residual diagnosis, consistent with the clinical and biochemical profile, was Liddle syndrome.

Genetic tests continue to hold the key to diagnosing this condition [18]. Targeted sequencing of the SCNN1B and SCNN1G genes would be both efficient and economical, considering that mutations in these subunits cause almost all of the cases reported. It would certainly be cheaper than exome sequencing, and this would make it preferable where there are budget limitations [19, 20, 22, 23].

But in this case, genetic tests were even economically unfeasible. A supervised trial with ENaC inhibitors, combined with biochemical monitoring, can be considered a diagnostic strategy where the budget is limited. The presence or absence of family history cannot be used to rule out the diagnosis since the condition is known to occur in a de novo fashion.

Traditional RAAS‐based treatments such as ACE inhibitors, ARBs, and mineralocorticoid receptor antagonists (like spironolactone) have little impact on Liddle’s syndrome patients since the underlying problem occurs at an earlier stage than aldosterone‐mediated signaling. The therapy for the disorder thus relies mainly on pharmacologically inhibiting ENaC using medications such as amiloride and triamterene [11].

In this particular patient, administration of amiloride (initially 5 mg/day, then uptitrated to 10 mg) resulted in an immediate and definite improvement of the symptoms. Although other medications (losartan and nebivolol) had been used previously during hospitalization, the patient’s systolic blood pressure exceeded 200 mmHg. Upon administration of amiloride, the pressure gradually decreased to 128/82 mmHg in 2 weeks, with normalization of serum potassium in the first week of therapy, which is a highly characteristic reaction observed both in previous studies and cases, wherein ENaC‐dependent hypertension was the most common clinical presentation [12, 21].

Discharge from the hospital was carried out using amiloride at a dose of 10 mg per day, in addition to losartan and nebivolol to mitigate the residual cardiovascular risks. Even though ARBs and beta‐blockers can be used together to reduce blood pressure, they do not counteract overactivation of ENaC, while mineralocorticoid receptor antagonists are only effective when aldosterone is responsible. Thus, future management will depend on sustained inhibition of ENaC activity, biochemical monitoring, dietary sodium reduction, and family cascade screening when possible.

This case highlights the importance of increased clinical suspicion for monogenic hypertension in adolescents suffering from severe hypertension with unknown causes of hypokalemia. The early detection of Liddle’s syndrome is clinically significant as a result of the fact that the specific inhibition of ENaC can quickly correct electrolyte disturbances and blood pressure, thus lowering the risk of complications associated with hypertension. In resource‐limited settings, the combination of clinical reasoning and treatment response to amiloride may be considered an alternative diagnostic approach in the absence of genetic testing.

## 4. Conclusion

The current case study emphasizes the significance of taking into account Liddle syndrome as one of the possible diagnoses in young patients presenting with severe hypertension, hypokalemia, and metabolic alkalosis, especially if the ratio of aldosterone to renin is within normal limits. Though left ventricular hypertrophy is a known complication of chronic and untreated hypertension, the current case study shows that a hypertensive crisis in a young patient must be a red flag, pointing at a need to evaluate underlying factors contributing to the condition. In countries with limited resources, where genetic testing is not available, administration of amiloride can be a helpful measure.

## Author Contributions

Abbas Khan and Alina Batool contributed to the conceptualization, initial draft, writing, methodology, and provision of resources. Askar Nisar and Jaweria Farman provided a critical review. Okasha Tahir, Muhammad Hassaan Javaid, and Raghabendra Kumar Mahato were responsible for revising the manuscript, offering detailed feedback aimed at improving content, editing, grammar, and clarity. Tayyeb Ali contributed to the initial draft and methodology.

## Funding

No funding was received for this manuscript.

## Disclosure

This study is not commissioned and externally peer reviewed.

## Ethics Statement

The authors have nothing to report.

## Consent

Written informed consent was obtained from the patient for publication of this case report and accompanying images. A copy of the written consent is available for review by the editor‐in‐chief of this journal on request.

## Conflicts of Interest

The authors declare no conflicts of interest.

## Data Availability

Data sharing is not applicable to this article as no datasets were generated or analyzed during the current study.

## References

[1] Liddle G. W. , Bledsoe T. , and Coppage W. S.Jr, A Familial Renal Disorder Simulating Primary Aldosteronism But With Negligible Aldosterone Secretion, Transactions of the Association of American Physicians. (1963) 76, 199–213.

[2] Tetti M. , Monticone S. , Burrello J. et al., Liddle Syndrome: Review of the Literature and Description of a New Case, International Journal of Molecular Sciences. (2018) 19, no. 3, 10.3390/ijms19030812. PMC5877673 29534496

[3] Findling J. W. , Raff H. , Hansson J. H. , and Lifton R. P. , Liddle’s Syndrome: Prospective Genetic Screening and Suppressed Aldosterone Secretion in an Extended Kindred, Journal of Cinical Endocrinology and Metabolism. (1997) 82, no. 4, 1071–1074, 10.1210/jcem.82.4.3862. 9100575

[4] Lu C. , Pribanic S. , Debonneville A. , Jiang C. , and Rotin D. , The PY Motif of ENaC, Mutated in Liddle Syndrome, Regulates Channel Internalization, Sorting and Mobilization from Subapical Pool, Traffic. (2007) 8, no. 9, 1246–1264, 10.1111/j.1600-0854.2007.00602.x. 17605762

[5] Bruce M. C. , Kanelis V. , Fouladkou F. , Debonneville A. , Staub O. , and Rotin D. , Regulation of Nedd4-2 Self-Ubiquitination and Stability by a PY Motif Located Within Its HECT-Domain, Biochemical Journal. (2008) 415, no. 1, 155–163, 10.1042/BJ20071708. 18498246

[6] Enslow B. T. , Stockand J. D. , and Berman J. M. , Liddle’s Syndrome Mechanisms, Diagnosis and Management, Integrated Blood Pressure Control. (2019) 12, 13–22, 10.2147/IBPC.S188869. 31564964 PMC6731958

[7] Wang L. P. , Yang K. Q. , Jiang X. J. et al., Prevalence of Liddle Syndrome Among Young Hypertension Patients of Undetermined Cause in a Chinese Population, Journal of Clinical Hypertension. (2015) 17, no. 11, 902–907, 10.1111/jch.12598. 26075967 PMC8031848

[8] Salih M. , Gautschi I. , van Bemmelen M. X. et al., A Missense Mutation in the Extracellular Domain of αENaC Causes Liddle Syndrome, Journal of the American Society of Nephrology. (2017) 28, no. 11, 3291–3299, 10.1681/asn.2016111163. 28710092 PMC5661275

[9] Cui Y. , Tong A. , Jiang J. , Wang F. , and Li C. , Liddle Syndrome: Clinical and Genetic Profiles, Journal of Clinical Hypertension. (2017) 19, no. 5, 524–529, 10.1111/jch.12949. 27896928 PMC8030933

[10] Rossier B. C. , Bochud M. , and Devuyst O. , The Hypertension Pandemic: An Evolutionary Perspective, Physiology. (2017) 32, no. 2, 112–125, 10.1152/physiol.00026.2016. 28202622

[11] Bhatt D. L. , Kandzari D. E. , O′Neill W. W. et al., A Controlled Trial of Renal Denervation for Resistant Hypertension, New England Journal of Medicine. (2014) 370, no. 15, 1393–1401, 10.1056/nejmoa1402670. 24678939

[12] Malde D. J. , Clarkson P. M. , and Tack C. J. , A Review of the Diagnosis and Management of Liddle Syndrome, Journal of Human Hypertension. (2020) 34, no. 5, 329–337.

[13] Rodby R. , Hypertension With Hypokalemic Metabolic Alkalosis: The Diagnosis is Apparent, Clinical Journal of the American Society of Nephrology. (2023) 18, no. 7, 965–968, 10.2215/CJN.0000000000000166. 37027796 PMC10356131

[14] Hundemer G. L. and Vaidya A. , Primary Aldosteronism Diagnosis and Management: A Clinical Approach, Endocrinology and Metabolism Clinics of North America. (2019) 48, no. 4, 681–700, 10.1016/j.ecl.2019.08.002. 31655770 PMC6824480

[15] Nair R. and Vaqar S. , Renovascular Hypertension, StatPearls. (2026) StatPearls Publishing, https://www.ncbi.nlm.nih.gov/books/NBK551587/. 31869068

[16] John T. A. and Anastasopoulou C. , Hypercortisolism (Cushing Syndrome), StatPearls, 2026, StatPearls Publishing, https://www.ncbi.nlm.nih.gov/books/NBK551526/. 31855370

[17] Pourian M. , Mostafazadeh D. B. , and Soltani A. , Does This Patient Have Pheochromocytoma? A Systematic Review of Clinical Signs and Symptoms, Journal of Diabetes and Metabolic Disorders. (2016) 15, 10.1186/s40200-016-0230-1. PMC4797176 26998444

[18] Enslow B. T. , Bhakta S. , Bhakta G. N. et al., Novel SCNN1B and SCNN1G Mutations Causing Liddle Syndrome With Diverse Clinical Presentations, Kidney International Reports. (2019) 4, no. 6, 877–886.31194187

[19] Monticone S. , Tetti M. , Buffolo F. et al., ENaC Is Not Part of the Aldosterone Signalling Pathway in Liddle Syndrome, Journal of Hypertension. (2018) 36, no. 1, 68–75.

[20] Kerstens M. N. , Kobold A. C. M. , Volmer M. et al., A Newly Identified Mutation in the Beta Subunit of the Epithelial Sodium Channel Causes Severe Refractory Hypertension, Journal of Hypertension. (2006) 24, no. 8, 1461–1466.

[21] Almajid A. N. , Patel P. , and Cassagnol M. , Amiloride, StatPearls, 2026, StatPearls Publishing, https://www.ncbi.nlm.nih.gov/sites/books/NBK542303/. 31194443

[22] Manas F. and Singh S. , Pseudohypoaldosteronism Type II or Gordon Syndrome: A Rare Syndrome of Hyperkalemia and Hypertension With Normal Renal Function, Cureus. (2024) 16, no. 1, 10.7759/cureus.52594. PMC10874887 38374860

[23] Chrysant S. G. and Luu T. M. , Effects of Amiloride on Arterial Pressure and Renal Function, Journal of Clinical Pharmacology. (1980) 20, no. 5-6 Pt 1, 332–337, 10.1177/009127008002000504. 7400369

